# A spatial national health facility database for public health sector planning in Kenya in 2008

**DOI:** 10.1186/1476-072X-8-13

**Published:** 2009-03-06

**Authors:** Abdisalan M Noor, Victor A Alegana, Peter W Gething, Robert W Snow

**Affiliations:** 1 Malaria Public Health and Epidemiology Group, Centre for Geographic Medicine, KEMRI – University of Oxford – Wellcome Trust Collaborative Programme, Kenyatta National Hospital Grounds (behind NASCOP), PO Box 43640-00100, Nairobi, Kenya; 2 Centre for Tropical Medicine, John Radcliffe Hospital, University of Oxford, Oxford, OX3 9DS, UK; 3 Spatial Ecology and Epidemiology Group, Tinbergen Building, Department of Zoology, University of Oxford, South Parks Road, Oxford, OX1 3PS, UK

## Abstract

**Background:**

Efforts to tackle the enormous burden of ill-health in low-income countries are hampered by weak health information infrastructures that do not support appropriate planning and resource allocation. For health information systems to function well, a reliable inventory of health service providers is critical. The spatial referencing of service providers to allow their representation in a geographic information system is vital if the full planning potential of such data is to be realized.

**Methods:**

A disparate series of contemporary lists of health service providers were used to update a public health facility database of Kenya last compiled in 2003. These new lists were derived primarily through the national distribution of antimalarial and antiretroviral commodities since 2006. A combination of methods, including global positioning systems, was used to map service providers. These spatially-referenced data were combined with high-resolution population maps to analyze disparity in geographic access to public health care.

**Findings:**

The updated 2008 database contained 5,334 public health facilities (67% ministry of health; 28% mission and nongovernmental organizations; 2% local authorities; and 3% employers and other ministries). This represented an overall increase of 1,862 facilities compared to 2003. Most of the additional facilities belonged to the ministry of health (79%) and the majority were dispensaries (91%). 93% of the health facilities were spatially referenced, 38% using global positioning systems compared to 21% in 2003. 89% of the population was within 5 km Euclidean distance to a public health facility in 2008 compared to 71% in 2003. Over 80% of the population outside 5 km of public health service providers was in the sparsely settled pastoralist areas of the country.

**Conclusion:**

We have shown that, with concerted effort, a relatively complete inventory of mapped health services is possible with enormous potential for improving planning. Expansion in public health care in Kenya has resulted in significant increases in geographic access although several areas of the country need further improvements. This information is key to future planning and with this paper we have released the digital spatial database in the public domain to assist the Kenyan Government and its partners in the health sector.

## Background

Accurate health information is the cornerstone of effective decision-making and reliable assessment of disease burden and resource needs [1-3]. Efforts to tackle the enormous burden of ill-health in low-income countries are hampered by the lack of functioning health information structures to provide reliable health statistics [4-10]. Central to a fully operational Health Information Systems (HIS) is a basic inventory of all functioning health facilities and the services they provide. Such an inventory requires a spatial dimension, allowing facilities to be linked to the populations they serve and other proximate determinants of health such as environment, poverty and education. This spatial linkage can be provided by geographic information systems (GIS). The use of GIS for health services planning is widespread in developed countries [11-13] but there are few examples of their development and operational use in resource poor settings in Africa [14-17].

In Kenya, a map of health facilities was produced in 1959 [18] but not up-dated until 2003 [15]. Since then, there has been an expansion in funding and resources in the health sector following the election of a new government in 2002 that promoted the establishment of a constituency development fund (CDF) to fund local development projects [19] including the building of new health centres and dispensaries where need was defined by the constituency. To reflect these changes and map the equity of expanded service provision since 2003, we have updated the spatial audit of public health facilities in Kenya against high resolution population density maps projected to 2008.

## Methods

### Developing a National Health Facility Database

In 2003 a national database of government, mission, non-governmental organization, local authority and private sector health service providers was completed and its assembly is presented in detail elsewhere [15]. In brief, all available health facility listings were reconciled to identify a single, comprehensive list and each facility provided with a unique code based on its location. Facilities were further coded by level of service provision (from hospital at the highest level through to dispensary at the lowest level) and by sector (Ministry of Health (MoH), Mission, Non-Government Organization (NGO) or private). Each facility was then geo-referenced using a variety of available national and district-level mapping sources including 1:50,000 scale topographical maps, on-screen digitized hand drawn maps from district-level reports, digital place names gazetteers and specialized surveys undertaken by research groups [15]. All information was exported to ArcView 3.2 (ESRI Inc., USA) and health facility maps and lists were sent to each District Health Management Team responsible for the over-sight of service provision in 69 second-level administrative areas to finalize checking and confirmation.

By 2007 there had been no concerted effort to update a single list of health service providers across Kenya with a unique spatial identifier. However, since the first facility audit and mapping exercise there have been several notable improvements in information available on both the existence of service providers and their location that could be used to update and improve the database constructed in 2003. These included improved commodity distribution lists used to audit the delivery of new anti-malarial drugs, anti-retroviral drugs and insecticide-treated nets by the MoH and its partners; a nationwide mapping project undertaken using global positioning systems (GPS) of all road and major public institutions close to these roads by the Ministry of Roads and Public Works (MRPW) [20]; and an increasing use of GPS to map district level institutions as part of research projects, development assistance programmes and a nationwide schools mapping project funded by the United States Agency for International Aid (USAID) managed by the Ministry of Education [21]. Finally, the launch of Google Earth in June 2005 [22] has meant that iteratively the imagery of Kenya has improved, allowing visualization of buildings and roads when streamed at high resolutions. This added capacity to locate structures in space has been used to triangulate crude coordinates provided from hand-drawn maps and other less reliable sources to visualize building structures likely to be health facilities and re-positioned more accurately. Over a period of three months in 2008 these combined sources were used to re-configure an updated inventory of health service providers and improve the resolution of geo-referencing. No new information was available on the private sector, a prolific and hard to enumerate source of health care in Kenya. The analysis presented here therefore considers only the public sector providers.

### Defining geographic access to public health services in Kenya

**Figure 1:**
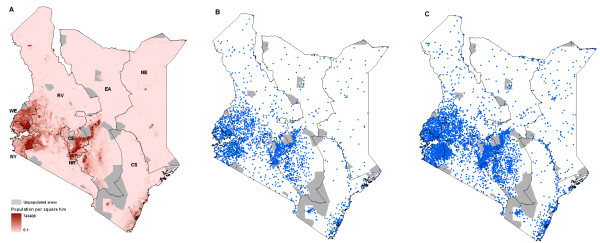
**Province maps of Kenya showing**: **A) **100 m resolution population density (Tatem et al 2007); **B) **distribution of public health facilities in 2003 (n = 3,048)*; and **C) **distribution of public health facilities in 2008 (n = 4,933)*. 390 health facilities that were not spatially positioned 67 specialist facilities that do not provide services to ambulatory patients are not shown on the both health facility maps. CE = Central province; CO = Coast province; EA = Eastern province; NE = North Eastern province; NR = Nairobi province; NY = Nyanza province; RV = Rift Valley; WE = Western province. *Health facilities that fall within unpopulated areas such parks and game reserves serve staff working in these establishments and/or communities around the protected areas.

According to the national health sector strategy for 1994 – 2010 a key benchmark for progress is that of geographic access to health services [23]. The strategy requires that all households in the country are located within 5 km, or a 1 hour travel time equivalent, to a public health facility. Here we have regarded health services as general out-patient care offering clinical services to ambulatory patients and have thus elected to exclude from this analysis the specialist health clinics that may only provide maternity services or specialized care for tuberculosis or mental illness. To compute access we have assessed the number of people within distance bands of each of each health service location in 2003 and 2008 using a 100 m × 100 m interpolated population density map (Figure 1A) described in detail elsewhere [24] and available from the Malaria Atlas Project (MAP) [25]. In brief, a combination of satellite imagery and land cover maps was used to develop models that identified the location of settlements [24,26]. The settlements map was used to redistribute census population counts within the small area polygons resulting in a population distribution map at 100 m × 100 m resolution. The accuracy of this population map was subsequently validated using actual data and was found to be significantly better than other gridded population products [24]. This raster 1999 population surface was projected to 2003 and 2008 using provincial inter-censal growth rates [27]. Euclidean (straight-line) distances were computed from each health facility to each population pixel at 100 m resolution for both 2003 and 2008 using ArcGIS 9.2 (ESRI Inc., USA). The continuous distance map was then classified into two distance bands: population within 5 km; and > 5 km from a public health facility and population counts in 2003 and 2008 extracted within each distance band.

## Results

The 2003 national health facility database recorded 3,512 public health facilities providing care to ambulatory patients including 232 hospitals; 694 health centres; 2,541 dispensaries; and 45 unspecified clinics (Table 1). The MoH managed 59% of these facilities; 33% by the mission/NGO sector and 3% by local authorities (Table 1). Following a careful triangulation of commodity distribution lists, revised health facility lists and facilities identified during the national roads mapping exercise, a total of 5,334 public health facilities providing care to ambulatory patients were identified as operational in 2008. Of these facilities the MoH was responsible for the management of 3,574 (67%), mission/NGO managed 1,484 (28%); and local authorities managed 101 (2%) while the remainder belonged to employers and other public institutions. A total of 40 unspecified clinics managed by employers and other ministries that were functioning in 2003 had closed by 2008.

**Table 1:** Public health facilities in Kenya in 2003 and 2008 by type and management agency.

	**Ministry of health**		**Mission/NGO**		**Local Authority**		**Employers and Other Ministries**		**Total**		
**Type of Facility**	**2003**	**2008**	**2003**	**2008**	**2003**	**2008**	**2003**	**2008**	**2003**	**2008**	**(% change)**
Hospital^b^	125	193	96	104	0	1	11	7	**232**	**305**	**24**
Health centre^c^	473	678	157	225	50	53	14	15	**694**	**971**	**29**
Dispensary	1,471	2,703	907	1,148	40	47	123	148	**2,541**	**4,046**	**37**
Unspecified clinic^d^	-	-	-	7	-	-	45	5	**45**	**12**	**-74**

**Total^e^**	**2,069**	**3,574**	**1,160**	**1,484**	**90**	**101**	**193**	**175**	**3,512**	**5,334**	**34**

a. 2003 data were derived from Noor et al 2004.

b. Includes provincial, district, sub-district hospitals or unspecified hospitals offering general inpatient clinical services. The 304 hospitals identified in 2004–08 included 50 health centres identified at the end of 2003 but were subsequently upgraded to hospitals.

c. Includes all health centers, sub-health centers, and rural health training centers as specified on national databases. The 975 health centres identified in 2004–08 included 187 (144 ministry of health; 41 mission/NGO; and 2 local authority) dispensaries identified at the end of 2003 but were subsequently upgraded to health centres.

d. Includes all clinics that were not classified in the private or employer sectors that provide generalized health services but were not classed as dispensaries or health centers.

e. 72 (in 2003) and 67 (in 2008) health facilities including all hospitals that provide treatment for special diseases only, such as leprosy, tuberculosis, cancer, ophthalmology, and spinal injury and the large number of maternity and nursing homes are not shown in this table and do not form part of subsequent analysis of geographic access to public health services as they do not provide care to ambulatory patients. The total of public health facilities also does not include 40 unspecified clinics managed by public institutions and non-MoH government ministries.

Overall 1,862 more public health facilities providing out-patient services were identified in 2008 compared to a similar exercise in 2003 (Table 1) representing an overall five-year increase of 35%. Among these new facilities, 1,690 (91%) were dispensaries, representing a 37% five-year increase in this lowest level of out-patient care nationwide (Table 1). Increases in hospital (24%) and health centre (29%) level care were less dramatic and largely a result of up-grading of existing facilities to new levels of service provision rather than new constructions. The largest increases in facilities were among those managed by the MoH (43% over five years) with far fewer being found in the Mission/NGO sector (23% growth over five years) (Table 1).

In 2003 we were unable to geo-locate 267 (15%) of public health facilities and 2,292 (64%) were positioned using crude sources such as hand-drawn maps or 1:50,000 topographical maps. Very few (21%, n = 693), were located using GPS sources (Noor et al 2004). Significant improvements have been made on the precise spatial positioning of both facilities identified in 2003 and new facilities established after 2003. Through a combination of mapping methods, 93% of the MoH and 90% of the mission/NGO facilities were spatially positioned in the 2008 database. GPS coordinates were used to identify 2,048 (38%) of public health facilities and only 314 (6%) and 215 (4.3%) were identified through the use of 1:50,000 maps hand-drawn maps, respectively. By 2008, 390 (7%) of the public health facilities remained un-positioned, which would require direct consultation with personnel at district levels or bespoke field surveys to locate.

**Figure 2:**
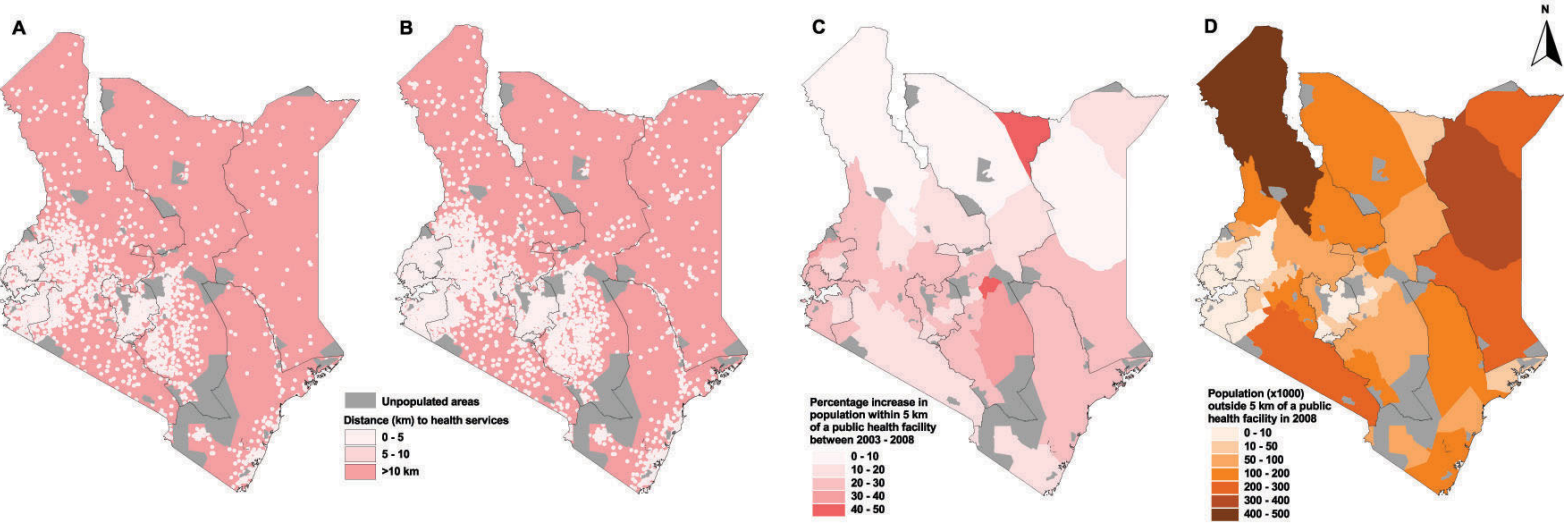
**Province maps of Kenya showing**: **A) **areas in 2003 that were within the 5 km benchmark of geographic access to public health services recommended by the ministry of health*; **B) **areas in 2008 that were within the 5 km benchmark of geographic access to public health services recommended by the ministry of health*; **C) **percentage increase in population within 5 km of a public health facility from 2003 to 2008**; and **D) **number of people in 2008 that were outside the 5 km benchmark to public health services***. Geographic access is represented as Euclidean (straightline) distance to public health facilities. 390 service providers that were not spatially positioned and all specialist facilities that do not provide services to ambulatory patients (72 in 2003 and 67 in 2008) were not included in the computation of distances to health facilities. *11% of the population was outside 5 km of a public health facility in 2008 compared to 29% in 2003. **The highest percentage increase in public health facilities in 2003–2008 occurred in areas in North Eastern province (Table 2). Most areas in this province, however, also registered the lowest proportional increase in population within 5 km of a public health facility in the same period. ***Several of the large, sparsely populated areas of northern part of the country had 100,000 or more people of the population outside of a 5 km of a public health facility accounting for 80% of the population in some of these areas. Areas within provinces in 2B and 2C represent the districts as at December 2007 (n = 69). Since then the number of districts have increased to 149 but the digital boundaries these new districts were not available at the time of the study.

The highest percentage increase between 2003 and 2008 in public health facilities occurred in North Eastern province (70%), followed by Nyanza (40%) and Eastern (39%) with Nairobi showing the lowest increase of less than a quarter (22%) (Table 2). Overall, 89% of the population was within the national target of 5 km distance to a public health facility in 2008 compared to 71% in 2003. This improvement, however, varied widely across the country (Figure 2). Although North Eastern province registered the highest growth in public health facilities (68%), it remained the least well served with only 29% of the population within 5 km of a public health service provider in 2008, an increase of about 10% from 2003 (Table 2 & Figure 2B &2C). Of the 4 million people who were outside the 5 km benchmark in 2008, approximately 3.3 million (83%) were from the northern, predominantly pastoralist, areas of the Rift Valley, North Eastern and Eastern provinces (Table 2 & Figure 2D).

**Table 2:** Provincial summaries of changes in number of public health facilities and population within the national target of 5 km of a public health service provider in 2003 and 2008.

**Province**	**Number of public health facilities in 2003**	**Number of public health facilities in 2008**	**% increase in public health facilities from 2003 to 2008**	**Population in 2003**	**Population within 5 km of a public health facility in 2003 (%)**	**Population in 2008**	**Population within 5 km of a public health facility in 2008 (%)**
Central	437	657	34	3,933,597	3,427,483 (82.0)	4,665,152	4,555,059 (97.6)
Coast	330	432	24	2,808,870	1,862,461 (66.3)	3,254,162	2,708,321 (83.2)
Eastern	600	992	40	5,144,073	3,233,429 (62.9)	5,882,336	5,103,168 (86.8)
Nairobi	211	272	22	2,596,918	2,211,329 (83.6)	2,962,125	2,961,837 (100.0)
North Eastern	53	164	68	1,319,338	237589 (18.0)	1,150,133	331,737 (28.8)
Nyanza	472	799	41	4,823,786	3,997,043 (80.8)	5,591,531	5,569,071 (99.6)
Rift Valley	1,134	1,615	30	7,950,358	4,962,210 (62.4)	9,266,543	7,523,223 (81.2)
Western	274	403	33	3,874,160	2,975,949 (76.8)	4,294,284	4,274,647 (99.5)

**Total**	**3,511**	**5,334**	**34**	**32,451,100**	** 22,907,493 (71.0)**	**37,066,266**	**33,027,063 (89.1)**

Population estimates were derived using inter-censal growth rates from the 1999 national census [citation [27]]

## Discussion

The Kenya health sector has experienced dramatic increases in the number of service providers over the last five years but their documentation, in terms of the number of health facilities and their location has been fragmented, programme specific, and difficult to integrate. It is difficult to imagine any effective service planning without an inventory of current providers spatially configure in relation to the distribution of the human population. Despite attempts in 2003 to reconcile all available information on the location, level and management of health services in Kenya [15] there remains no centralized authoritative spatially-defined inventory of health service providers in 2008. The 2003 database was provided to the MoH in 2004 with the intention that the resource would serve as the platform for future planning. In the absence of an updated, centralized database we have repeated the audit of service providers in 2008. We estimate that there has been a 34% expansion in public health facilities providing out-patient care since 2003, the majority supported by the MoH.

The construction of new health facilities has led to significant improvement in geographic access by patients to service providers from 71% of population within the national target of 5 km of public health service providers in 2003 to 89% in 2008. In spite of these improvements, sparsely populated, predominantly pastoralist, areas in the north of the country contained between 100,000 and 500,000 people outside of the required geographic access to public health care (Figure 2C) and accounted for over 80% of the approximately 4 million people living beyond 5 km from a public health service provider in 2008. It is unlikely that any construction of new health facilities will yield significant improvements in geographic access in Central, Nairobi, Nyanza and Western provinces, all of which have 97% to 100% of their population within 5 km of a public health facility. The Coast, Eastern and Rift Valley provinces already have more than 80% of their populations with appropriate geographic access to public health care and only a few strategically located additional health service providers are required to increase access, particularly in their remote sparsely populated reaches. North Eastern province, however, lags considerably behind other provinces in terms of geographic access and significant investment in new health service providers is required to bridge this gap.

In addition to monitoring geographic access, the applications of a spatially referenced inventory of service providers are manifold because they can be linked to other geo-referenced datasets. For example we have shown elsewhere that where only a minority of health facilities report their health statistics, these can be used, together with the full dataset, and with appropriate geo-statistical methods, to estimate complete disease burden coverage with acceptable accuracies at national and provincial levels [28,29].Such approaches have been adapted to examine temporal changes in out-patient malaria burdens [30] and the estimation of national anti-malarial drug requirements [31]. At management levels a facility inventory should provide the basis for financial planning and monitoring, stock management, personnel deployment and regular quality assurance sampling. Whether modeling disease risks or planning commodity distribution it is critical that sources of data or recipients of drugs are positioned spatially.

The development of a database of health service providers is a continuous process and its long-term success depends on a number of technical, financial and management issues. In this study, it became clear that the quality and completeness of existing health facility lists are driven by programme-specific interests to the extent that key departments within the Ministry of Health rely on different facility lists, each believed to be the 'universe' of health services in the country. In compiling the lists into a single database a major challenge has been the lack of a consistent naming of health facilities and the absence of a unique and common health facility code making data integration difficult. In addition, although 93% of the public health facilities in the 2008 database are now spatially-referenced, only 38% were mapped using 'gold-standard' GPS method, albeit an 18% increase from those in 2003. Given that most of the facilities are located in the densely populated middle belt of the country (Figure 1) and are located close to each other, it is important that the proportion of facilities mapped using GPS is increased to ensure they can be linked accurately to the populations they serve using newly available high resolution maps [24].

With publication of this paper we have released all the spatially configured data we have been able to assemble into the public domain. It is available as a database with longitude and latitude and along with metadata describing how these were established [25] along with KMZ files suitable for export to Google Earth [22]. The MoH is currently up-grading its facility listings [32], however this inventory does not have facility coordinates and cannot be used in any mapping or spatial analysis. We hope that the release of our geo-referenced data will assist those concerned with the measurement of equity and service provision. This database should serve as a template for future work and we still recommend a nationwide census of public health service providers, similar to the national school mapping exercise sponsored by the USAID [21], to serve as a confirmatory exercise and improve the quality-of-care and human resource attribute data necessary to transform a population-to-provider platform into a truly valuable planning tool. An efficient system for regular updating of the health service database also needs to be put in place by the Ministry of Health. Possibilities of expanding such an exercise to the important but poorly regulated and prolific private health sector in Kenya must be explored.

## Abbreviations

CDF: Constituency Development Fund; GPS: Global positioning system; HIS: Health information system; KEMRI: Kenya Medical Research Institute; LA: Local Authority; MAP: Malaria Atlas Project; MoH: Ministry of Health; MRPW: Ministry of Roads and Public Works; NGO: Non-governmental organization; USAID: United States Agency for International Aid.

## Competing interests

The authors declare that they have no competing interests.

## Authors' contributions

AMN was responsible for study design, data cleaning, analysis, interpretation and production of the final manuscript. VAA contributed to data cleaning and analysis and production of final manuscript. PWG contributed to data, analysis, interpretation and production of the final manuscript. RWS was responsible for overall scientific management, analysis, interpretation and preparation of the final manuscript.

## Funding source

This study received financial support from The Wellcome Trust, UK (#058922), Roll Back Malaria Initiative, AFRO (AFRO/WHO/RBM # AF/ICP/CPC/400/XA/00); Management Sciences for Health (MSH: Cooperative Agreement #GHN-A-00-07-00002-00) and the Kenya Medical Research Institute. AMN is supported by the Wellcome Trust as a Research Training Fellow (#081829). RWS is supported by the Wellcome Trust as Principal Research Fellow (#079080). Both AMN and RWS acknowledge the support of the Kenyan Medical Research Institute.

## References

[B1] Lippeveld T Routine Health Information Systems: the glue of a unified health system. Keynote address at the workshop on issues and innovation in routine health information in developing countries, Potomac, 14–16 March 2001.

[B2] Detmer D (2003). Building the national health information infrastructure for personal health, health care services, public health, and research. BMC Med Inf Decis Making.

[B3] WHO 60th World Health Assembly, Resolution 60.27 Strengthening of health information systems. http://www.who.int/gb/ebwha/pdf_files/WHA60/A60_R27-en.pdf.

[B4] Osiobe S (1989). Health information imperatives for third world countries. Soc Sci Med.

[B5] Attaran A (2005). An Immeasurable Crisis? A Criticism of the Millennium Development Goals and Why They Cannot Be Measured. PLoS Med.

[B6] Lopez AD, Mathers CD, Ezzati M, Jamison DT, Murray CJL (2006). Global Burden of Disease and Risk Factors. Disease Control Priorities Project.

[B7] Braa J, Humberto M (2007). Building collaborative networks in Africa on health information systems and open source software development – Experience from the HISP/BEANISH network.

[B8] Stansfield S, Walsh J, Prata N, Evans T (2006). Information to Improve Decision Making for Health. 1,017–1,030. Disease Control Priorities in Developing Countries.

[B9] Boerma JT, Stansfield SK (2007). Health statistics now: are we making the right investments?. Lancet.

[B10] WHO (2008). The Health Metrics Network Framework.

[B11] Bullen N, Moon G, Jones K (1996). Defining localities for health planning: a GIS approach. Soc Sci Med.

[B12] Gatrell AC, Markku L, Anthony C, Gatrell, Markku, Loytonen (1998). GIS and health.

[B13] Hewitt A, Tinline R (2004). GIS and health care: spatial accessibility of cancer clinics in Hamilton, Ontario.

[B14] Booman M, Sharp BL, Martin CL, Manjate B, La Grange JJ, Durrheim DN (2003). Enhancing malaria control using a computerised management system in southern Africa. Malaria J.

[B15] Noor AM, Gikandi PW, Hay SI, Muga RO, Snow RW (2004). Creating spatially defined databases for health service planning in resource poor countries: The example of Kenya. Acta Trop.

[B16] Kazembe LN, Appleton CC, Kleinschmidt I (2007). Geographical disparities in core population coverage indicators for Roll Back Malaria in Malawi. Int J Equity Health.

[B17] Lozano-Fuentes S, Elizondo-Quiroga D, Farfan-Ale JA, Loroño-Pino MA, Garcia-Rejon J, Gomez-Carro S, Lira-Zumbardo V, Najera-Vazquez R, Fernandez-Salas I, Calderon-Martinez J, Dominguez-Galera M, Mis-Avila P, Morris N, Coleman M, Moore CG, Beaty BJ, Eisen L (2008). Use of Google Earth™ to strengthen public health capacity and facilitate management of vector-borne diseases in resource-poor environments. Bull World Health Organ.

[B18] Butler RJ Atlas of Kenya: A comprehensive series of new and authenticated maps prepared from the national survey and other government sources with gazetteer and notes on pronunciation and spelling. The Survey of Kenya 1959, Nairobi, Kenya.

[B19] Consituency Development Fund. http://www.kippra.org/Constituency.asp.

[B20] Ministry of Roads and Public Works (2004). Classified Digital Road Network in Kenya.

[B21] Ministry of Education Inception Report. Consultancy on development of a GIS database of learning Institutions (School mapping exercise), 2008.

[B22] Google Earth http://earth.google.com/.

[B23] Ministry of Health (1994). Health Policy Framework 1994–2010.

[B24] Tatem AJ, Noor AM, von Hagen C, di Gregorio A, Hay SI (2007). High resolution population maps for low income nations: combining land cover and census in East Africa. PLoS One.

[B25] Malaria Atlas Project, Data. http://www.map.ox.ac.uk/MAP_data.html.

[B26] Tatem AJ, Noor AM, Hay SI (2004). Defining approaches to settlement mapping for public health management in Kenya using medium spatial resolution satellite imagery. Rem Sens Environ.

[B27] Central Bureau of Statistics (2001). 1999 population and housing census: counting our people for development. Population distribution by administrative areas and urban centres.

[B28] Gething PW, Noor AM, Gikandi PW, Ogara EAA, Hay SI, Nixon MS, Snow RW, Atkinson PM (2006). Improving Imperfect Health Management Information System Data in Africa Using Space-Time Geostatistics. PLoS Med.

[B29] Gething PW, Noor AM, Gikandi P, Hay SI, Nixon MS, Atkinson P (2007). Developing geostatistical space-time models to predict malaria outpatient treatment burdens in Kenya. Geogr Analysis.

[B30] Gething PW, Noor AM, Goodman CA, Gikandi P, Hay SI, Sharif SK, Atkinson P, Snow RW (2007). Information for decision making from imperfect national data: tracking major changes in health care use in Kenya using geostatistics. BMC Med.

[B31] Gething PW, Noor AM, Okiro EA, Mutheu JJ, Alegana VA, Hay SI, Memusi D, Amin AAm Tetteh G, Snow RW (2008). Defining Medicine and Commodity Needs for the Management of Uncomplicated and Severe Malaria in Kenya's Formal Sector Using Novel Space-Time Geostatistical Methods. Submitted to the US Agency for International Development by the Strengthening Pharmaceutical Systems Program.

[B32] Kenya Health Facilities. Version 1.0.25. http://www.kenyahealthfacilities.net/voxiva_prd/tmp/sc_xls_20090126031144_181_facility_grid_view.xls.

